# Ethical dilemmas in the care of patients suffering from psychotic catatonia: a case report

**DOI:** 10.3389/fpsyt.2025.1543563

**Published:** 2025-02-05

**Authors:** Luigi F. Saccaro, Barbara Privé, Ambra D’Imperio, Claire Bridel, Othman Sentissi, Alexandre Wullschleger

**Affiliations:** ^1^ Psychiatry Department, Faculty of Medicine, University of Geneva, Geneva, Switzerland; ^2^ Psychiatry Department, Geneva University Hospital, Geneva, Switzerland; ^3^ Forensic Psychiatry and Psychology Department, Bern University Hospital, Bern, Switzerland; ^4^ Department of Preclinical Medicine, Institute of History and Ethics in Medicine, TUM School of Medicine and Health, Technical University of Munich, Munich, Germany; ^5^ Department of Medicine, Faculty of Medicine, University of Geneva, Geneva, Switzerland

**Keywords:** catatonia, ethics, MRI, case report, psychiatry, psychosis, nonvoluntary treatment, Weddinger Model

## Abstract

**Background:**

Coercive measures in psychiatric practice are controversial due to their potential for severe negative effects. Ethical debates focus on respecting autonomy, minimizing damaging effects, and acting in the patient’s best interest.

**Case:**

We present a unique case of a young patient suffering from a first episode of catatonic psychosis, in which striking this balance was especially difficult given the patient’s complete mutism and opposition, given the absence of immediate danger to herself or others, the lack of anamnestic information, and her avoidance of social support, which would have meant that she would not have encountered psychiatric care, were it not for an exceptional government plan in place at the time of hospitalization. The patient showed a very favorable, persistent response to nonvoluntary treatment with haloperidol and lorazepam, which could be discussed and debriefed once she recovered, after almost 5 months of hospitalization and follow-up.

**Discussion:**

Arguments for and against nonvoluntary treatment are reviewed based on discussion with the local ethics committee, providing a useful reference for future similar cases. Finally, this case highlights an atypical onset of psychosis in a previously high-functioning individual and explores the mental health impact of international tensions, particularly the Russian-Ukrainian war, on individuals.

## 1 Introduction

Nonvoluntary measures in psychiatric practice, including nonvoluntary medication, nonvoluntary hospitalization, seclusion or mechanical restraint, have long been a topic of debate. Nonvoluntary treatment refers to interventions undertaken when a patient, although unable to provide explicit informed consent due to a mental health crisis, is believed to have previously expressed values or preferences that justify the intervention. Such measures respect the patient’s presumed wishes and prior rational values, which may have been compromised by the mental illness. In contrast, involuntary treatment is imposed on individuals who are deemed to lack decision-making capacity, are coerced, or pose an imminent danger to themselves or others, irrespective of their prior preferences or expressed values. While involuntary treatment is often justified by immediate safety concerns, nonvoluntary treatment acknowledges the ethical importance of aligning interventions with the patient’s historical values and goals, even in the absence of current consent (1).

Both nonvoluntary and involuntary measures are associated with significant ethical challenges and potential adverse effects. Patients subjected to these interventions are at an elevated risk of nonvoluntary or involuntary readmissions, and often report feelings of shame, distrust, and perceptions of punishment or abuse (1–4). Additionally, these measures in general have a detrimental effect on the therapeutic relationship. Although involuntary or nonvoluntary measures are considered a last resort when personal or public safety is at immediate risk, determining when to apply them in clinical practice can be challenging. The complexity is heightened in cases where there is not an immediate threat to safety, but severe neglect and worsening health conditions outside of emergency situations exist (5). Ethics committees can play a critical role in nonvoluntary and involuntary psychiatric interventions by mitigating bias, promote transparency, and provide accountability by independently reviewing cases, balancing autonomy, nonmaleficence, justice, and beneficence (6), and safeguarding against the misuse of coercive measures (7). Their involvement is vital in complex cases where ethical and clinical considerations intersect.

Indeed, the ethical debate surrounding these issues centers on balancing patient autonomy with the duty of care (8, 9). Autonomy, a key principle of medical ethics, grants individuals the right to make their own healthcare decisions. However, in psychiatry, mental illness can impair decision-making capacity, complicating the application of this principle (10). These concerns arise particularly when there is uncertainty regarding the patient’s capacity for self-determination, both in cognitive and volitional aspects, as well as ambiguity surrounding the existence of risk and the criteria justifying nonconsensual treatment. While involuntary or nonvoluntary measures override personal autonomy, this is sometimes necessary to act in the patient’s presumed best interests, particularly when the patient lacks insight into their condition. In this sense, the principle of beneficence—acting for the patient’s well-being—can support the use of nonvoluntary treatment to prevent harm or deterioration. Striking a balance between autonomy, nonmaleficence, and beneficence requires careful case-by-case consideration, guided by ethical and legal frameworks designed to protect patient’s rights. Open dialogue, patient involvement in decisions where possible, and robust safeguards are crucial for navigating these ethical challenges and ensuring that nonvoluntary treatment is used judiciously and ethically. In Switzerland, nonvoluntary medication is uniquely regulated to ensure a separate assessment of consent for treatment, distinct from involuntary hospitalization. Article 434 of the Civil Code governs the provision of treatment without consent, even in non-emergency situations. This legislation permits the involuntary administration of medication in three circumstances: 1) when a patient poses a serious risk to themselves or others, 2) when the patient cannot recognize the need for treatment, and 3) when no less invasive alternatives exist to mitigate the risk (5).

Efforts to reduce coercion have led to various interventions, including de-escalation training, improved ward infrastructure, and patient-initiated advance plans (11–13). These strategies have proven effective, resulting in integrated programs aimed at reducing coercion and promoting patient-centered, recovery-oriented care. An example is the Weddinger Model (14), which emphasizes patient involvement, individualized care, and multidisciplinary collaboration. However, implementing such models requires effective communication with patients, their families, and a comprehensive understanding of their desires and social context. This is particularly challenging in cases of psychotic disorders—the most common diagnosis in involuntarily hospitalized patients (15)—especially when catatonia is present. Here, the concept of supported decision-making becomes crucial, as do tools like crisis plans and advance directives, which can help ensure that the patient’s preferences are respected as much as possible.

Psychosis and catatonia are two distinct yet interconnected psychiatric phenomena. Psychosis, often characterized by hallucinations, delusions, and disorganized thinking and behavior, can occur in various psychiatric conditions, such as schizophrenia, and severely impair a patient’s personal and social functioning, besides their somatic health (16). Catatonia, on the other hand, is a complex, potentially life-threatening neuropsychiatric syndrome commonly characterized by immobility, mutism, posturing, stereotypies, negativism, and echolalia, although at least forty separate signs of catatonia have been described (17). Affecting up to 10% of psychiatric inpatients, catatonia can occur in association with psychiatric disorders, such as schizophrenia, or as a result of medical conditions, including autoimmune encephalitis, neurodegenerative disorders, or metabolic disturbances (17, 18). Notably, the presence of catatonia in schizophrenia patients correlates with heightened mortality rates, accentuating the critical importance of effective understanding and management (17). In clinical settings, high-dose lorazepam or other benzodiazepines remain the first-line therapy for catatonia, and the Bush Francis Catatonia Rating Scale is currently the most sensitive and best validated rating instrument for broad clinical use with acutely catatonic patients (17, 19). While the relationship between psychosis and catatonia is not fully understood, they frequently overlap, with catatonic symptoms often emerging alongside or during psychotic episodes.

We present below a case report unique in several aspects. First, it highlights an unusual ethical dilemma involving the nonvoluntary hospitalization and treatment of a non-aggressive, non-self-harming patient who would not have come into contact with psychiatric care if not for an exceptional plan enacted by local authorities at the time of hospitalization, as described below. Second, the case demonstrates a favorable response to a typical antipsychotic, which is uncommon in catatonic patients with psychotic disorders (17, 19). Third, it describes an atypical and relatively late presentation of psychosis in a previously high-functioning individual. Lastly, the case underscores the impact of international tensions, specifically the Russian-Ukrainian war, and social pressures on the mental health of individuals.

## 2 Case presentation

**Figure 1:**
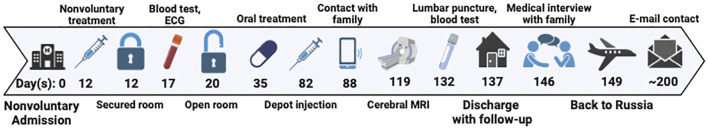
Timeline of clinical events. This figure summarizes the key events in the patient’s clinical history from the time of admission onward.

A 31-year-old Russian woman was involuntarily admitted to our Adult Psychiatry Acute Ward (Day 0, **Figure 1**) as part of a national plan offering emergency shelter during a particularly cold winter. Under this plan, individuals who refused shelter despite life-threatening outdoor temperatures were assessed in the emergency room(ER) for their capacity for discernment. Those deemed capable of choosing to continue sleeping outdoors were discharged. Since our patient was not communicating and did not exhibit somatic symptoms, she was involuntarily hospitalized in our ward for a serious state of neglect.

Upon admission, the patient appeared in generally good health, with appropriate hygiene and winter clothing. She was normovigilant, calm, but oppositional, refusing physical examinations, ECG, or blood tests. She exhibited no reaction when touched to empty her pockets. Her eye contact was absent, and she would close her eyes or cover her face with her hat. She displayed withdrawal behaviors and stereotypies, such as rocking back and forth. There were no signs of substance intoxication or withdrawal. Due to her mutism, her mood and cognition could not be evaluated, though she appeared relaxed and sometimes smiled. The patient stayed mostly in her room or the garden, avoiding contact with others. A diagnosis of catatonic syndrome (characterized by mutism, opposition, stereotypies, and withdrawal) was made, with a Bush-Francis score of 11/69, which has very good sensitivity and specificity when more than two screening signs are present, as in this case (20, 21).

For two weeks, the patient’s clinical condition remained largely unchanged. She continued to refuse interaction, examination, or treatment (such as lorazepam), despite explanations provided in various languages, including her native language through an interpreter. Her sleep and appetite appeared normal.

Local social workers had known the patient for several years, citing her complete mutism and opposition as obstacles to assisting her. Despite this, she managed to live independently in a tent, which was well-organized with valuable electronics (e.g. a professional camera, iPad), and personal care items. Interpol searches and contact with the Russian embassy or local police yielded no new information. However, an online search revealed she held a PhD and had previously worked as a postdoctoral researcher at a Northern European university.

On Day 14, after consulting the hospital ethics committee, recurrent nonvoluntary treatment was initiated (haloperidol 5 mg up to 2x/day orally, PO, or intramuscular, IM, if refused, and lorazepam 2.5 mg 3x/day PO or 4 mg IM if refused). In this case, haloperidol was chosen as the primary antipsychotic due to its proven efficacy, injectable form, availability in both the local setting and the patient’s country of origin. When offered PO treatment, the patient remained mute with her eyes and mouth closed, prompting an IM injection of 4 mg lorazepam and 5 mg haloperidol, which she did not resist. Thirty minutes later, however, she attempted to flee the ward, trying to open a window forcefully, all while remaining mute. Given her unpredictable behavior and the unassessable risk of self-harm or aggression, her room was secured, and constant supervision was initiated. After four days of treatment, the patient accepted an ECG and blood tests for the first time, which were overall unremarkable, showing no metabolic disorders. She began displaying improved interaction (smiling at caregivers) and ate with other patients. However, she exhibited new strange behaviors such as riding a stationary bike backwards, walking in circles around the smoking shelter, keeping plastic bags on her feet, covering her room’s ventilation with plastic bags, and pulling out electrical wires.

On Day 20, the patient spoke for the first time during a medical interview in her room. Her speech was in English, laconic and only when prompted, but was overall coherent without blockages. Her thought process was normal, her mood neutral, and she appeared relaxed, smiling, even using humor, without major signs of catatonia. She denied having suicidal thoughts, though she expressed a non-systematic persecutory delusion, believing her country was spying on her through hidden cameras. She was also completely anosognosic. Seclusion and constant supervision were lifted, though she accepted PO treatment for the first time only on Day 35. After one week of oral treatment, the patient regressed to her admission state (mute, lying in bed, refusing to eat with others, displaying odd behaviors). She was frequently absent in the afternoons but returned for dinner, refusing to explain her unauthorized outings. Suspecting non-compliance with oral medication, as she refused a blood test to assess haloperidol levels, we reverted to IM treatment, as above.

On Day 52, the patient opened up about her personal history for the first time. She reported being divorced for 4 years before admission in our ward, without children. After working as a postdoctoral researcher in a large extraeuropean city, she had relocated internationally three years before admission. About two years before admission, she had resigned from her position, believing that individuals, allegedly sent by her research advisor and parents in Russia, were controlling her to force her into marriage and childbearing. She had used her savings to attend scientific conferences but eventually ran out of money, living on the streets for about two years before her admission. Despite no clinical history of psychiatric disorders or substance abuse, she had maintained delusional ideas of persecution with an interpretative and intuitive mechanism, particularly involving her family and research advisor. On Day 52, the patient accepted PO treatments as above, on the condition that the haloperidol dose be reduced to 7.5 mg (in oral solution to improve compliance). On Day 54, she agreed to meet with social workers to discuss discharge planning. However, on Day 77, due to progressive clinical deterioration (return to being withdrawn, laconic, with a PANSS score of 111/120) and suspected non-compliance, systematic IM haloperidol 10 mg daily was re-introduced. By Day 82, her PANSS score had improved to 71/120, and she accepted a monthly depot injection of haloperidol Decanoas 100 mg.

On Day 88, the patient contacted her mother for the first time since admission. While she feared returning to Russia, she agreed to let her mother visit her in the hospital. During the meeting on Day 95, her treatment plan and the diagnosis of schizophrenia with catatonia were discussed, with persistent delusional disorder as a potential differential diagnosis. The patient expressed a medium-term goal of applying for refugee status and finding a job, with hopes of eventually returning to her scientific career.

On Day 105, the patient agreed for the first time to an MRI brain scan, recognizing it as standard care for her first psychotic episode. By Day 119, the MRI revealed mild parietal, and to a lesser extent, fronto-temporal atrophy. Neurological examination revealed only the presence of an inexhaustible nasopalpebral reflex, and a lumbar puncture was recommended, in agreement with the patient, given also that a report from the ER three years before admission indicated that she had sought treatment for tick removal and was not mute at the time. An extensive blood and cerebrospinal fluid workup revealed no evidence for an autoimmune encephalitis, a neurodegenerative or infectious condition, or a prion disease. Brain atrophy resulting from a probable perinatal insult was hypothesized.

The patient was discharged on Day 137 after the civil court decided to end her nonvoluntary hospitalization. She was provided with emergency housing, social worker support, a scheduled follow-up neurological consultation, psychiatric follow-up with a mobile team, and monthly haloperidol Decanoas injections.

Two weeks after discharge, the patient reported during a psychiatric interview at her apartment that she had remained mute due to a belief that healthcare providers were connected to the police, fearing arrest due to her Russian nationality and the war in Ukraine. However, she now criticized these persecutory ideas and expressed gratitude towards the medical team. She affirmed that the medication helped her feel safe and trust others. Her psychiatric status was normal, without psychotic elements, and she expressed a desire to return to Russia to rejoin her family. During a second meeting the following day, the patient and her family rediscussed with the psychiatrist the clinical documents they received, the diagnosis, and the need for antipsychotic treatment and psychiatric follow-up, which they planned to continue in Russia.

Approximately two months after discharge, the patient emailed the hospital, reporting that she was doing well and requested her complete biological and neuroimaging results for her clinical follow-up in Russia.

## 3 Discussion

This case presents several ethical implications regarding the treatment of the non-aggressive patient with psychosis and catatonic symptoms. The decision of nonvoluntary hospitalization was made in an exceptional setting instructed by government authorities, on the assumption that whoever refused emergency shelter to avoid the severe meteorological conditions should be assessed for their capacity to make such a potentially life-threatening choice. The absence of elements to determine such capacity and our patients’ mutism spurred the ER doctors to hospitalize her.

Once hospitalized, the decision to initiate nonvoluntary pharmacological treatment had to be considered. Arguments against this course of action included: 1) the absence of evident signs of self-harm or danger to others, 2) the patient’s non-verbal refusal of treatment, 3) the potentially traumatic nature and risks associated with nonvoluntary treatment, in line with the principle of non-maleficence, 4) the fact that the patient had been living independently, albeit under difficult circumstances, suggesting she had managed her daily life to some extent, 5) the impossibility to conduct necessary examinations to rule out risk factors for adverse effects of pharmacological treatment, or to identify alternative causes of the symptomatology, before treatment initiation.

After consulting the hospital ethics committee, the following arguments for nonvoluntary treatment were gathered. 1) The patient presented a rapid and significant decline from her previous high-functioning state as a university postdoctoral researcher few years before, to a state of neglect, isolation, and homelessness having lived on the streets for more than a year. 2) Her symptoms, particularly catatonia and mutism, were compatible with serious, time-sensitive psychiatric conditions that become increasingly difficult to treat the more they are left untreated (22). 3) Due to the aforementioned symptoms, she likely lacked discernment regarding necessary care, raising concerns related to the principle of justice (6), which ensures that individuals, even those unable to advocate for themselves due to their mental and social conditions, are not denied appropriate treatment. 4) Without treatment, her physical and mental health were at serious risk of further deterioration, especially in the context of a suspected worsening psychosis and, more generally, of severe outdoor weather conditions besides the risk of victimization living on the street (in this context, the treatment agreed with the principle of beneficence based on the need for timely intervention to prevent further deterioration). 5) Her previous decision to seek medical attention for tick removal years prior, when her mental state had not yet visibly declined, suggested that her deterioration was relatively rapid and progressive in the year preceding her hospitalization. 6) Finally, she did not flee the hospital during the first weeks, even though her room and the ward were open, via the easily accessible garden, nor did she leave once the treatment began to take effect.

Providing a definitive answer is beyond the scope of this article, which aims at fostering ethical discussion and the ongoing efforts towards reducing as much as possible nonvoluntary measures. Strengths of the present article include the longitudinal clinical assessments, the extended time span of clinical observation, the comprehensive somatic examinations to exclude alternative explanations for the symptoms, and adherence to the CARE checklist (**Supplementary Materials**). While some case reports on psychiatric patients presenting with mutism exist (e.g. in the context of malingering, schizophrenia, or dissociative disorders), ethical implications are not their focus and they could rely on significant anamnestic information (23, 24). In agreement with existing literature, our patient’s catatonia responded very well to treatment with lorazepam and, interestingly, her psychotic symptoms demonstrated a favorable response to a typical antipsychotic, which is uncommon in catatonic patients with psychotic disorders (17, 19). Limitations are related to the specific characteristics of the clinical case. Despite our best effort, reconstruction of the clinical history in the years before admission was limited, in part due to the patient’s own partial memories of that period. Similarly, PANSS scoring for the initial part of the hospitalization is lacking. Secondly, given the rapid return of the patient to Russia, we do not have access to long-term follow-up. However, the fact that she required complementary clinical information by email and that she wanted to return to Russia and her family is reassuring, suggesting that the persecutory delusions were and remained controlled, and that the patient engaged actively in her clinical follow-up in Russia. Additionally, the patient’s perspective, as expressed in the last interviews, also shows insight and understanding of her clinical history, and a detailed debriefing of the nonvoluntary measures could be done with the patient and her family.

In conclusion, the ethical implications of nonvoluntary treatment in non-aggressive patients with psychosis and catatonic symptoms demand a careful consideration of beneficence, and the patient’s individual circumstances. This highlights how acute impairments in discernment, affecting both the volitional and cognitive components of decision-making, may increase the risk of malpractice, particularly in cases involving nonvoluntary treatment. However, such interventions may result in significant long-term improvement and the restoration of self-determination, especially when approached through multidisciplinary collaboration guided by ethical principles.

## Data Availability

The original contributions presented in the study are included in the article/**Supplementary Material**. Further inquiries can be directed to the corresponding author.

## Supplementary material

Supplementary Material CARE checklist.

## References

[1] Sisti DA. Nonvoluntary psychiatric treatment is distinct from involuntary psychiatric treatment. JAMA. (2017) 318:999–1000. doi: 10.1001/jama.2017.10318 28837717

[2] Chieze M Hurst S Kaiser S Sentissi O. Effects of seclusion and restraint in adult psychiatry: A systematic review. Front Psychiatry. (2019) 10. doi: 10.3389/fpsyt.2019.00491 PMC6673758 31404294

[3] Sibitz I Scheutz A Lakeman R Schrank B Schaffer M Amering M. Impact of coercive measures on life stories: qualitative study. Br J Psychiatry. (2011) 199:239–44. doi: 10.1192/bjp.bp.110.087841 21778173

[4] Hirsch S Thilo N Steinert T Flammer E. Patients’ perception of coercion with respect to antipsychotic treatment of psychotic disorders and its predictors. Soc Psychiatry Psychiatr Epidemiol. (2021) 56:1381–8. doi: 10.1007/s00127-021-02083-z PMC8316198 33904940

[5] Meroni G Sentissi O Kaiser S Wullschleger A. Treatment without consent in adult psychiatry inpatient units: a retrospective study on predictive factors. Front Psychiatry. (2023) 14:1224328. doi: 10.3389/fpsyt.2023.1224328 37636826 PMC10447976

[6] Beauchamp TL Childress JF. Principles of Biomedical Ethics. Oxford University Press (2001). 470 p.

[7] Vollmann J. Clinical ethics committees and ethics consultation in psychiatry. In: Helmchen H Sartorius N, editors. Ethics in Psychiatry: European Contributions. Springer Netherlands, Dordrecht (2010). p. 109–25. doi: 10.1007/978-90-481-8721-8_7

[8] Chieze M Clavien C Kaiser S Hurst S. Coercive measures in psychiatry: A review of ethical arguments. Front Psychiatry. (2021) 12:790886. doi: 10.3389/fpsyt.2021.790886 34970171 PMC8712490

[9] Wullschleger A Vandamme A Mielau J Heinz A Bermpohl F Mahler L. Relationship between perceived coercion and perceived justification of coercive measures – secondary analysis of a randomized-controlled trial. BMC Psychiatry. (2023) 23. doi: 10.1186/s12888-023-05192-y PMC10546675 37784077

[10] Appelbaum PS. Clinical practice. Assessment of patients’ competence to consent to treatment. N Engl J Med. (2007) 357:1834–40. doi: 10.1056/NEJMcp074045 17978292

[11] Celofiga A Kores Plesnicar B Koprivsek J Moskon M Benkovic D Gregoric Kumperscak H. Effectiveness of de-escalation in reducing aggression and coercion in acute psychiatric units. A cluster randomized study. Front Psychiatry. (2022) 13:856153/full. doi: 10.3389/fpsyt.2022.856153/full 35463507 PMC9021532

[12] de Jong MH Kamperman AM Oorschot M Priebe S Bramer W van de Sande R. Interventions to reduce compulsory psychiatric admissions: A systematic review and meta-analysis. JAMA Psychiatry. (2016) 73:657–64. doi: 10.1001/jamapsychiatry.2016.0501 27249180

[13] Malhotra C Shafiq M Batcagan-Abueg APM. What is the evidence for efficacy of advance care planning in improving patient outcomes? A systematic review of randomised controlled trials. BMJ Open. (2022) 12:e060201. doi: 10.1136/bmjopen-2021-060201

[14] Oster A Cole C Mahler L. The Weddinger Modell – A systematic review of the scientific findings to date and experiences from clinical practice. Med Res Arch. (2021) 9. doi: 10.18103/mra.v9i8.2521

[15] Chatzisymeonidis S Kioskli K. Insights and risk factors of involuntary hospitalizations through a retrospective analysis of police records: differences between involuntarily and non-hospitalized patients. Curr Psychol. (2023) 43:1–10. doi: 10.1007/s12144-023-04841-5

[16] Saccaro LF Aimo A Panichella G Sentissi O. Shared and unique characteristics of metabolic syndrome in psychotic disorders: a review. Front Psychiatry. (2024) 15:1343427. doi: 10.3389/fpsyt.2024.1343427 38501085 PMC10944869

[17] Penland HR Weder N Tampi RR. The catatonic dilemma expanded. Ann Gen Psychiatry. (2006) :5:14. doi: 10.1186/1744-859X-5-14 16959040 PMC1578553

[18] Rasmussen SA Mazurek MF Rosebush PI. Catatonia: Our current understanding of its diagnosis, treatment and pathophysiology. World J Psychiatry. (2016) 6:391–8. doi: 10.5498/wjp.v6.i4.391 PMC5183991 28078203

[19] Caroff SN Ungvari GS Gazdag G. Treatment of schizophrenia with catatonic symptoms: A narrative review. Schizophr Res. (2024) 263:265–74. doi: 10.1016/j.schres.2022.11.015 36404216

[20] Aandi Subramaniyam B Muliyala KP Suchandra HH Reddi VSK. Diagnosing catatonia and its dimensions: Cluster analysis and factor solution using the Bush Francis Catatonia Rating Scale (BFCRS). Asian J Psychiatr. (2020) 52:102002. doi: 10.1016/j.ajp.2020.102002 32506001

[21] Bush G Fink M Petrides G Dowling F Francis A Catatonia I. Rating scale and standardized examination. Acta Psychiatr Scand. (1996) 93:129–36. doi: 10.1111/j.1600-0447.1996.tb09814.x 8686483

[22] Marshall M Lewis S Lockwood A Drake R Jones P Croudace T. Association between duration of untreated psychosis and outcome in cohorts of first-episode patients: A systematic review. Arch Gen Psychiatry. (2005) 62:975–83. doi: 10.1001/archpsyc.62.9.975 16143729

[23] Aggarwal A Sharma DD Kumar R Sharma RC. Mutism as the presenting symptom: three case reports and selective review of literature. Indian J Psychol Med. (2010) 32:61–4. doi: 10.4103/0253-7176.70542 PMC3137816 21799563

[24] Graham PJ Foreman DM. An ethical dilemma in child and adolescent psychiatry. Psychiatr Bull. (1995) 19:84–6. doi: 10.1192/pb.19.2.84

