# Supplementary material for: Ethical dilemmas in the care of patients suffering from psychotic catatonia: a case report
Source: Front Psychiatry. 2025 Feb 5;16:1543563. doi: 10.3389/fpsyt.2025.1543563 (PMC11835877; doi:10.3389/fpsyt.2025.1543563)
Supplement: Supplementary Material — CARE checklist. [file DataSheet1.pdf]

CARE
case report guidelines

CARE Checklist of information to include when writing a case report

(cc) BY-NC-ND

Topic	Item	Checklist item description	Reported on Line
Title	1	The diagnosis or intervention of primary focus followed by the words "case report"	1
Key Words	2	2 to 5 key words that identify diagnoses or interventions in this case report, including "case report"	40
Abstract (no references)	3a	Introduction: What is unique about this case and what does it add to the scientific literature?	33-36
	3b	Main symptoms and/or important clinical findings	25-32
	3c	The main diagnoses, therapeutic interventions, and outcomes	25-32
	3d	Conclusion—What is the main "take-away" lesson(s) from this case?	33-36
Introduction	4	One or two paragraphs summarizing why this case is unique (**may include references**)	103-111
Patient Information	5a	De-identified patient specific information.	115-116
	5b	Primary concerns and symptoms of the patient.	117-121
	5c	Medical, family, and psycho-social history including relevant genetic information	167-176
	5d	Relevant past interventions with outcomes	167-176
Clinical Findings	6	Describe significant physical examination (PE) and important clinical findings.	123-155
Timeline	7	Historical and current information from this episode of care organized as a timeline	Section 2, Figure 1
Diagnostic Assessment	8a	Diagnostic testing (such as PE, laboratory testing, imaging, surveys).	123-155, 189-197
	8b	Diagnostic challenges (such as access to testing, financial, or cultural)	116-155, 217-270
	8c	Diagnosis (including other diagnoses considered)	184-186
	8d	Prognosis (such as staging in oncology) where applicable	208-213
Therapeutic Intervention	9a	Types of therapeutic intervention (such as pharmacologic, surgical, preventive, self-care)	143-182
	9b	Administration of therapeutic intervention (such as dosage, strength, duration)	198-210
	9c	Changes in therapeutic intervention (with rationale)	161-166
Follow-up and Outcomes	10a	Clinician and patient-assessed outcomes (if available)	202-213
	10b	Important follow-up diagnostic and other test results	189-213
	10c	Intervention adherence and tolerability (How was this assessed?)	176-182, 202-213
	10d	Adverse and unanticipated events	NA
Discussion	11a	A scientific discussion of the strengths AND limitations associated with this case report	251-270
	11b	Discussion of the relevant medical literature **with references**.	255-261, 72-111
	11c	The scientific rationale for any conclusions (including assessment of possible causes)	233-261, 189-197
	11d	The primary "take-away" lessons of this case report (without references) in a one paragraph conclusion	272-279
Patient Perspective	12	The patient should share their perspective in one to two paragraphs on the treatment(s) they received	263-270, 183-188, 202-213
Informed Consent	13	Did the patient give informed consent? Please provide if requested	Yes ☒ No ☐
